# A spatio-temporal analysis of scrub typhus and murine typhus in Laos; implications from changing landscapes and climate

**DOI:** 10.1371/journal.pntd.0009685

**Published:** 2021-08-25

**Authors:** Tamalee Roberts, Daniel M. Parker, Philip L. Bulterys, Sayaphet Rattanavong, Ivo Elliott, Koukeo Phommasone, Mayfong Mayxay, Vilada Chansamouth, Matthew T. Robinson, Stuart D. Blacksell, Paul N. Newton

**Affiliations:** 1 Lao-Oxford-Mahosot-Hospital-Wellcome Trust Research Unit, Microbiology Laboratory, Mahosot Hospital, Vientiane, Lao PDR; 2 Centre for Tropical Medicine & Global Health, Nuffield Department of Medicine, University of Oxford, United Kingdom; 3 University of California, Irvine, California, United States of America; 4 Department of Pathology, Stanford University, California, United States of America; 5 Institute of Research and Education Development (IRED), University of Health Sciences, Ministry of Health, Vientiane, Lao PDR; 6 Mahidol-Oxford-Research Unit, Faculty of Tropical Medicine, Mahidol University, Bangkok, Thailand; Seoul National University College of Medicine, REPUBLIC OF KOREA

## Abstract

**Background:**

Scrub typhus (ST) and murine typhus (MT) are common but poorly understood causes of fever in Laos. We examined the spatial and temporal distribution of ST and MT, with the intent of informing interventions to prevent and control both diseases.

**Methodology and principle findings:**

This study included samples submitted from 2003 to 2017 to Mahosot Hospital, Vientiane, for ST and MT investigation. Serum samples were tested using IgM rapid diagnostic tests. Patient demographic data along with meteorological and environmental data from Laos were analysed.

Approximately 17% of patients were positive for either ST (1,337/8,150 patients tested) or MT (1,283/7,552 patients tested). While both diseases occurred in inhabitants from Vientiane Capital, from the univariable analysis MT was positively and ST negatively associated with residence in Vientiane Capital. ST was highly seasonal, with cases two times more likely to occur during the wet season months of July-September compared to the dry season whilst MT peaked in the dry season. Multivariable regression analysis linked ST incidence to fluctuations in relative humidity whereas MT was linked to variation in temperature. Patients with ST infection were more likely to come from villages with higher levels of surface flooding and vegetation in the 16 days leading up to diagnosis.

**Conclusions:**

The data suggest that as cities expand, high risk areas for MT will also expand. With global heating and risks of attendant higher precipitation, these data suggest that the incidence and spatial distribution of both MT and ST will increase.

## Author summary

These data from Laos strongly support the hypothesis that murine typhus and scrub typhus are more urban and rural diseases, respectively. Risks of scrub typhus were associated with visiting rice fields and forests, with surface flooding and higher vegetation density. Risks of murine typhus were living in Vientiane City and not having visited rice fields and forests. Homes of patients with scrub typhus were consistently further away from the city centre and from roads than for those of patients with murine typhus. With the outward spread of higher house density with city expansion during the study, murine typhus spread outwards whilst scrub typhus receded. Temporal trends in incidence were associated, for murine typhus, with higher temperature and, for scrub typhus, with higher rainfall, humidity and flooding. In areas where both diseases are found, these data suggest that residence, occupational and climatic differential risks are useful for assisting with identifying patients. The data also suggest that as cities further enlarge, high risk areas for murine typhus will expand. With global heating and risks of attendant higher precipitation, these data also suggest that the incidence and spatial distribution of both murine typhus and scrub typhus will increase.

## Introduction

Scrub typhus and murine typhus are common causes of fever throughout Asia and are endemic in the Lao People’s Democratic Republic (Laos) [1,2]. Scrub typhus (ST) is caused by the bacterium *Orientia tsutsugamushi*, transmitted by the bite of larval trombiculid mites (“chiggers”) (*Leptotrombidium* spp.). Globally there are an estimated ~1 million cases of ST a year with a median untreated mortality of 6% [3]). Murine typhus (MT) has a much wider geographical distribution, known across all continents except Antarctica, but without estimates of global disease incidence [4]. MT is caused by the bacterium *Rickettsia typhi* and is transmitted by fleas, principally the rat flea *Xenopsylla cheopis* [4]. Both diseases are neglected with underestimated burdens on health, due in part to nonspecific symptoms and poor access to reference diagnostic assays. Although they can be easily treated with doxycycline, they result in severe disease and mortality in a significant minority [4].

Laos is a lower-middle income, land-linked country in South-East Asia with a dry season from November to May, and a wet season from late May to October. The hottest months are April and May, and the coolest are December and January. The 2015 Lao census reported a population of 6,492,228 [5], predominantly rural (67%). The urban population had increased by 6% since 2005 and the population density is highest in Vientiane Capital (209 people per square kilometre).

The first published description of diverse rickettsial diseases in Laos was of patients presenting to Mahosot Hospital with suspected community-acquired septicaemia, predominantly from Vientiane Capital and Vientiane Province; 14.8% of patients were positive for ST while 9.6% were positive for MT [2]. A subsequent seroprevalence study in Vientiane Capital in 2006 found high and comparable IgG seroprevalence of ST and MT (20.3% and 20.6%, respectively) [6]. However, the spatial distribution of seroprevalence differed, with ST seropositivity higher on the outskirts of Vientiane Capital, and MT seropositivity concentrated in the central urban areas. Similar findings have emerged from subsequent studies [1,7,8]. These findings suggest that ST is more common in rural areas than MT, but that there exists a significant burden of both diseases in urban areas. Laos, and in particular Vientiane Capital, is relatively unusual with both diseases sympatric and common.

Little is known about the temporal dynamics of these diseases, and the influence of climatic and environmental factors. Here we present the results from a 14-year study on ST and MT in Laos to improve our understanding of disease risks and inform interventions. For both diseases, we investigated individual level demographic and behavioural characteristics of patients; spatial and temporal distributions; meteorological correlates of the temporal patterns; and environmental and geographic correlates of the spatiotemporal distributions. We hypothesise that over time patients diagnosed with scrub typhus and murine typhus would have homes progressively farther away from Vientiane Capital, as improved transport links gave patients easier access to the diagnostic facilities at Mahosot Hospital and as the urban environment of Vientiane spreads into the surrounding countryside. We also hypothesise that temporal trends influence the incidence of both diseases in Laos.

## Methods

### Ethics statement

Ethical approval was obtained from the Oxford Tropical Research Ethics Committee (006–07) and from the Lao National Ethics Committee for Health Research. All patients gave written informed consent for participation. Written consent was obtained from the parent/guardian of each participant under 16 years of age. In Lao heath care, people aged 16 years and above are considered adults for health care decisions and the approved Lao national ethics clearance for this study included that patients aged 16 years and above are considered adults and therefore parent/guardian consent was not required.

### Clinical samples

Sera collected from patients with suspected typhus infection were collected between 2003 and 2017 and tested for scrub typhus and murine typhus IgM. Patient and demographic information were collected and analysed along with geographic, meteorological and environmental data from Laos to describe predictors of scrub typhus and murine typhus disease.

We included all sera collected from consenting inpatients, who the responsible physician suspected as having typhus infection, characterized by a minimum of fever, headache, and/or myalgia, between May 2003 and October 2017 sent to the Microbiology Laboratory at Mahosot Hospital, Vientiane City (17°57’36.2”N 102°36’43.3”E) for diagnostic workup. Anti-IgM antibody against *Orientia tsutsugamushi* was detected using the Panbio Scrub Typhus IgM and IgG Rapid cassette Test (Panbio, Australia) from 2003-June 2008, AccessBio CareStart Scrub Typhus IgM (AccessBio) kit from June 2008-April 2016 and the InBios Scrub Typhus Detect IgM Rapid Test (InBios International Inc., Seattle WA, USA) from April 2016 onwards. For *Rickettsia typhi* anti-IgM antibodies, the GenBio ImmunoDot (GenBio, USA), adapted for IgM (see [9]), was used and considered positive when three or more test dots were positive.

### Demographic, geographic, and environmental data

Patient admission demographic data, including age, sex, and home village were recorded. Patient home villages were georeferenced using official village coordinates from the Lao census [10].

Meteorological data for Vientiane Capital were exported from TuTiempo [11], including daily measurements of minimum, maximum, and mean temperature (^o^C), precipitation (mm), humidity (%), visibility (km), wind speed (km/h), and maximum sustained wind speed (km/h).

Environmental indices (EI) for surface water and vegetation were extracted from Moderate Resolution Imaging Spectroradiometer (MODIS) products (MOD13Q1/MYD13Q1 250 meter AQUA/TERRA 16 day composites). A normalized flooding index (NDFI) [12], the normalized differential vegetation index (NDVI), and the enhanced vegetation index (EVI) were all extracted for this analysis. NDFI gives an indication of surface water, NDVI gives an indication of surface vegetation, and EVI is an improvement on NDVI in that it is less sensitive to atmospheric conditions and forest canopies (S1 Document). The data were downloaded for each 16-day time interval (from June 2003–December 2017) using a 5km buffer around the home villages of each patient in the dataset.

Summary measures of the EIs were calculated for each home village listed in the data, including the mean and variance of NDFI, NDVI, and EVI across the entire study period. EIs that correspond to the 16 day period leading up to each patients’ hospital admission date were also included in this analysis.

Other environmental, demographic and geographic variables included the distance from each patients’ home village to the nearest highway (using OpenStreetMaps road data, S1 Document), village population, elevation and location.

### Exploratory analysis

The case incidence for each district and province was calculated as the number of diagnosed infections per 10,000 or 100,000 people per unit of time. Population data came from the 2005 and 2015 Lao censuses [5,13]. Univariable and multivariable logistic regressions were used to calculate odds ratios (OR) and model adjusted odds ratios for factors associated with ST or MT infections. Incomplete data were dropped from the multivariable analysis. Variables included: sex, having visited a rice field or forest in the last two weeks, having seen a rat in the last two weeks, having a cat at home, living in Vientiane Capital and having seen a flea in the last two weeks (specific to MT).

### Identification of meteorological predictors of scrub typhus and murine typhus infections

We performed regression analyses to identify meteorological factors associated with ST and MT. Case counts were aggregated by month for the entire study period to allow adequate sample sizes to detect associated factors. Meteorological factors were also summed (precipitation) or averaged (temperature, humidity, visibility, and wind speed) by month. Univariable and multivariable negative binomial regressions were performed (to account for over dispersion of count data), with meteorological factors as the independent variables and number of ST or MT cases as the outcome of interest. Variables significantly associated with incidence of either disease were examined simultaneously in multivariable models.

### Logistic regression for seasonal and geographic predictors of scrub typhus and murine typhus infections

Generalized additive logistic regression models were used to assess correlations between predictor variables and the odds of testing positive for either ST or MT, for all patients with successfully georeferenced home villages (total of 5760 out of 8701). Covariates included in the model are listed in S1 Table, and included the EIs (NDFI and EVI), year of hospital admission, seasonality (day of year), patient’s home village population, elevation, location and distance to the nearest major road. All covariates were included as smoothed terms in the models, and separate models were run for ST and for MT.

Linear regressions were used to assess potential changes in the distance of patient home villages from Vientiane City and to the nearest major road (S1 Document).

### Software

The data were analysed using Stata version 14 (StataCorp, College Station, TX) and R version 3.3.1 (using the MASS package for the negative binomial regressions and the *mgcv* package for generalized additive models). Maps were created using QGIS version 3.4.9. All layers were created by the authors of this manuscript.

## Results

### Patient information

#### Scrub typhus

Of 8,150 patients tested for ST between May 2003 and October 2017, 1,337 (16%) were IgM seropositive, with median age of 29 years and a slight predominance of males (58%). The data from all patients were included in the study although denominators vary for different parameters due to incomplete data. In the two weeks prior to symptom onset, 54% of ST patients (313/583) had visited a rice field, 85% (681/798) had seen a rat and 51% (287/559) had visited a forest (p<0.001, Table 1). From the multivariable analysis, ST was positively associated with having visited a rice field (OR 1.66, CI 1.25–2·11, p = 0.001) and having visited a forest (OR 3.06, CI 2.28–4.11, p<0.001) in the two weeks prior to symptom onset (Table 1). Most patients (67%, 878/1319) came from Vientiane Capital. Analysing only patients living in the more urban Vientiane Capital, similar relationships were found (S2 Table).

**Table 1:** Univariable and multivariable logistic regression for factors associated with scrub typhus positive patients from all patients tested for scrub typhus by RDT. n = number of patients with feature, N = number of patients with result (%). The data from all individual patients were included in the study although denominators vary for different parameters due to incomplete data.

Factor	Admission features for all patients tested for scrub typhus (8,150 patients) n/N (%)	Admission features of scrub typhus positive patients (1,337 patients) n/N (%)	Univariable analysis			Multivariable analysis		
			OR	95% CI	p-value	OR	95% CI	p-value
Age median (range): years	28 (1 day- 97 years)	29 (1 day- 90 years)	-	-	-	-	-	-
Sex	Male 4,498 (56), female 3,498 (44)	Male 760 (58), female 560 (42)	0.94	0.83–1.06	0.289	1.01	0.78–1.31	0.953
Visited a rice field in last 2 weeks	500/1,241 (40)	313 /583 (54)	**2.92**	**2.31–3.69**	**<0.001**	**1.66**	**1.25–2.11**	**0.001**
Visited a forest in last 2 weeks	423/1,210 (35)	287/559 (51)	**4.0**	**3.11–5.14**	**<0.001**	**3.06**	**2.28–4.11**	**<0.001**
Seen rat in last 2 weeks	3,223/3,506 (92)	681/798 (85)	**0.38**	**0.30–0.49**	**<0.001**	0.84	0.61–1.17	0.302
Cat at home	2,086/3,903 (53)	429/816 (53)	0.96	0.82–1.11	0.574	0.77	0.59–1.00	0.054
Lives in Vientiane Capital	5,533/7,762 (71)	878/1,319 (67)	**0.76**	**0.67–0.87**	**<0.001**	0.78	0.58–1.05	0.097

#### Murine typhus

Of 7,552 patients tested for MT between March 2004 and October 2017, 1,283 (17%) were IgM seropositive for MT. The median age was 33 years and there was a slight predominance of males (53%). During the two weeks prior to symptom onset, 15% (100/653) of patients had seen a flea, 86% (732/850) had seen a rat, 26% (175/657) visited a rice field and 17% (113/648) visited a forest (Table 2). Most patients (79%, 987/1254) came from Vientiane Capital. From the multivariable analysis, MT was positively associated with a home address in Vientiane Capital (OR 2.63, CI 1.86–3.72, p<0.001) and negatively associated with having visited a rice field (OR 0.64, CI 0.45–0.90, p = 0.011) or a forest (OR 0.322, CI 0.22–0.47, p<0.001) within the two weeks prior to symptom onset (Table 2). Seventy-nine (1%, 79/7001) patients were rapid diagnostic test (RDT) positive for both ST and MT.

**Table 2:** Univariable and multivariable logistic regression for factors associated with murine typhus positive patients for all patients tested for murine typhus by RDT. n = number of patients with feature, N = number of patients with result (%). The data from all individual patients were included in the study although denominators vary for different parameters due to incomplete data.

Factor	Admission features for all patients tested for murine typhus (7,552 patients) n/N (%)	Admission features of murine typhus positive patients (1,283 patients) n/N (%)	Univariable analysis			Multivariable analysis		
			OR	95% CI	p-value	OR	95% CI	p-value
Age median (range): years	24 (1 day- 96 years)	33 (20 days- 88 years)	-	-	-	-	-	-
Sex	Male 4,157 (56), 3,230 (44)	Male 676 (53), female 605 (47)	**1.19**	**1.05–1.34**	**0.005**	1.05	0.76–1.45	0.765
Visited a rice field in last 2 weeks	375/1,016 (37)	175/657 (26)	**0.29**	**0.22–0.38**	**<0.001**	**0.64**	**0.45–0.90**	**0.011**
Visited a forest in last 2 weeks	301/999 (30)	113/648 (17)	**0.18**	**0.14–0.25**	**<0.001**	**0.322**	**0.22–0.47**	**<0.001**
Seen rat in last 2 weeks	3,014/3,260 (92)	732/850 (86)	**0.35**	**0.27–0.45**	**<0.001**	1.27	0.85–1.88	0.239
Seen flea in last 2 weeks	660/1,498 (44)	100/653 (15)	**0.09**	**0.07–0.12**	**<0.001**	0.81	0.52–1.26	0.353
Cat at home	1934/3,557 (54)	535/945 (57)	1.13	0.97–1.31	0.106	1.27	0.92–1.76	0.142
Lives in Vientiane Capital	5,110/7,148 (72)	987/1,254 (79)	**1.59**	**1.37–1.84**	**<0.001**	**2.63**	**1.86–3.72**	**<0.001**

### Spatial and temporal distributions

**Fig 1:**
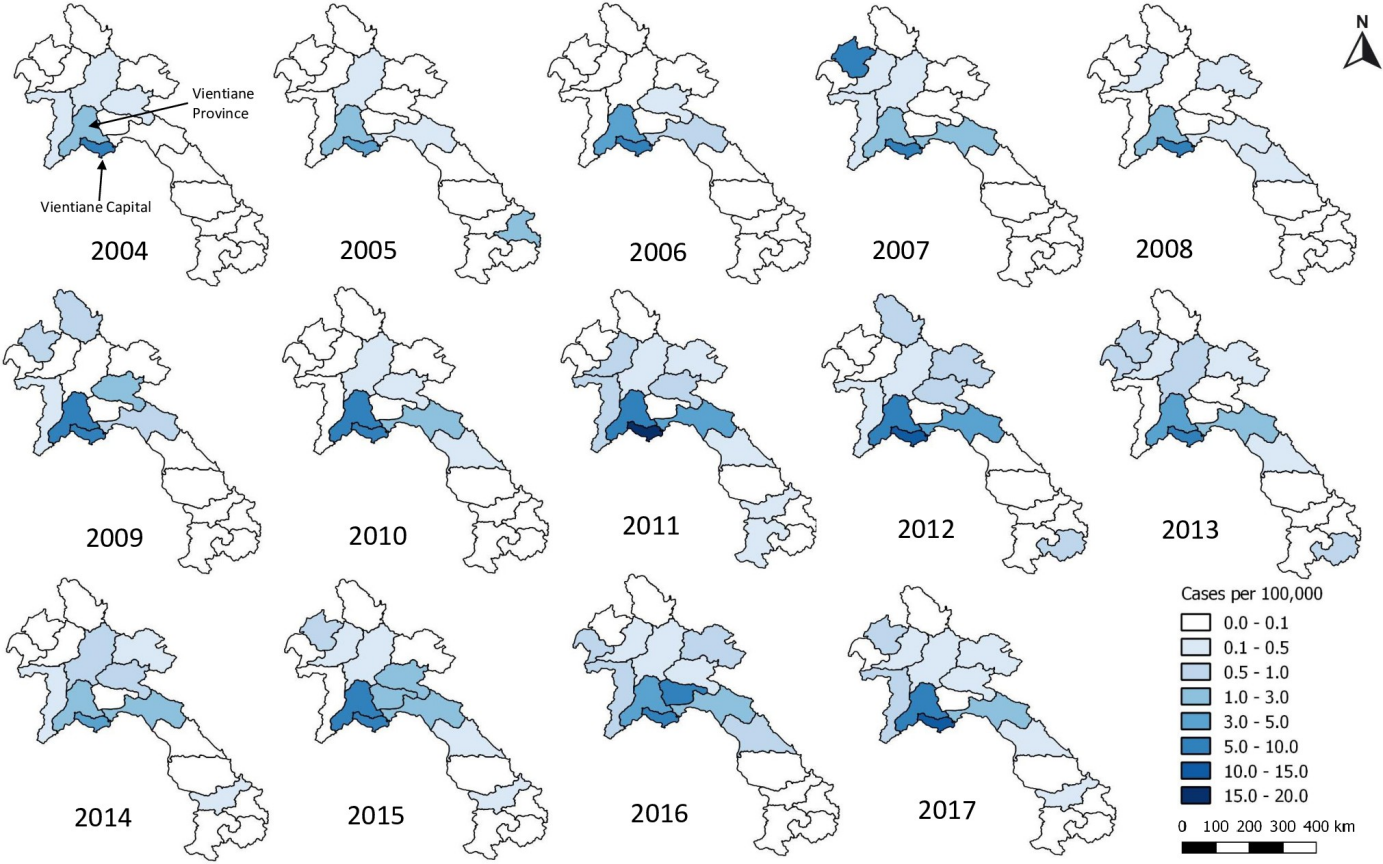
Patients with scrub typhus per 100,000 people per province in Laos from 2004–2017. Maps were created using QGIS version 3.4.9. All layers were created by the authors of this manuscript.

**Fig 2:**
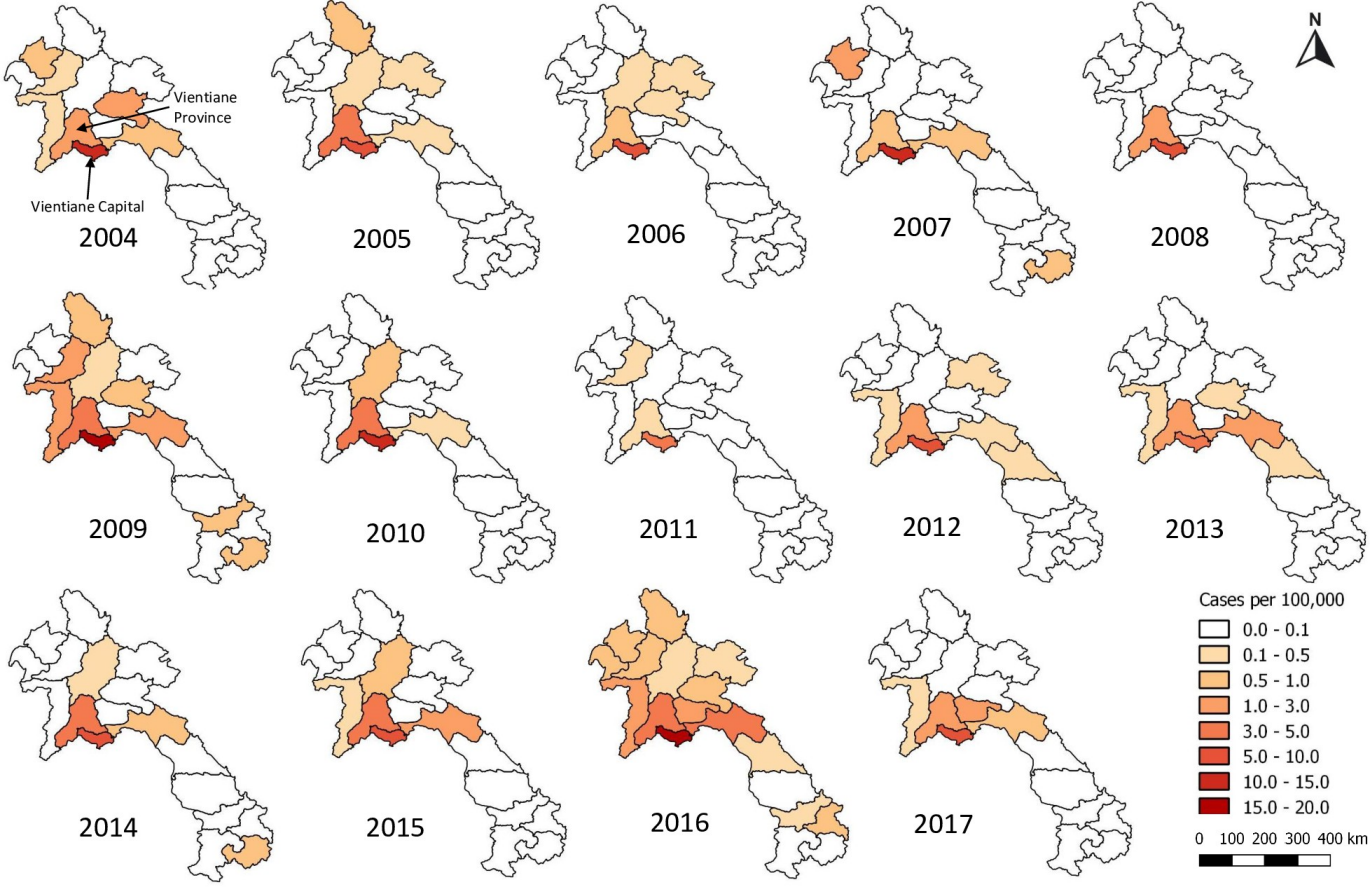
Patients with murine typhus per 100,000 people per province in Laos from 2004–2017. Maps were created using QGIS version 3.4.9. All layers were created by the authors of this manuscript.

Most patients with both diseases most commonly came from Vientiane Capital and then from Vientiane Province across all years (Figs 1 and 2). Sikhottabong District, Vientiane Capital, showed the greatest combined burden of ST and MT inpatients of all districts, with 207 (17.1/10,000 people) ST and 205 (16.9/10,000 people) MT. However, the highest incidence of ST patients admitted to hospital was from Naxaithong District (17.7/10,000 people), which is a peri-urban district, and the highest incidence of MT was in Sisattanak and Chanthabuly Districts (both 20.2/10,000 people), which are more urban districts. For Vientiane Capital and Vientiane Province, almost all patients came from villages that are situated along major roads (median distance to a major road for all patients’ homes was 460 meters; S1 Fig).

Patients came from increasingly farther from Vientiane City and from home villages farther from major roads over the study period. For each one year increase there was a corresponding increase in ST/MT patient home distance from Vientiane City of 850 meters and increase in distance from a major road of 68 meters (S3 Table). Patients diagnosed with ST had homes consistently farther away from Vientiane City (~19km) and from major roads (~1km; S4 Table) in comparison to MT patients.

**Fig 3:**
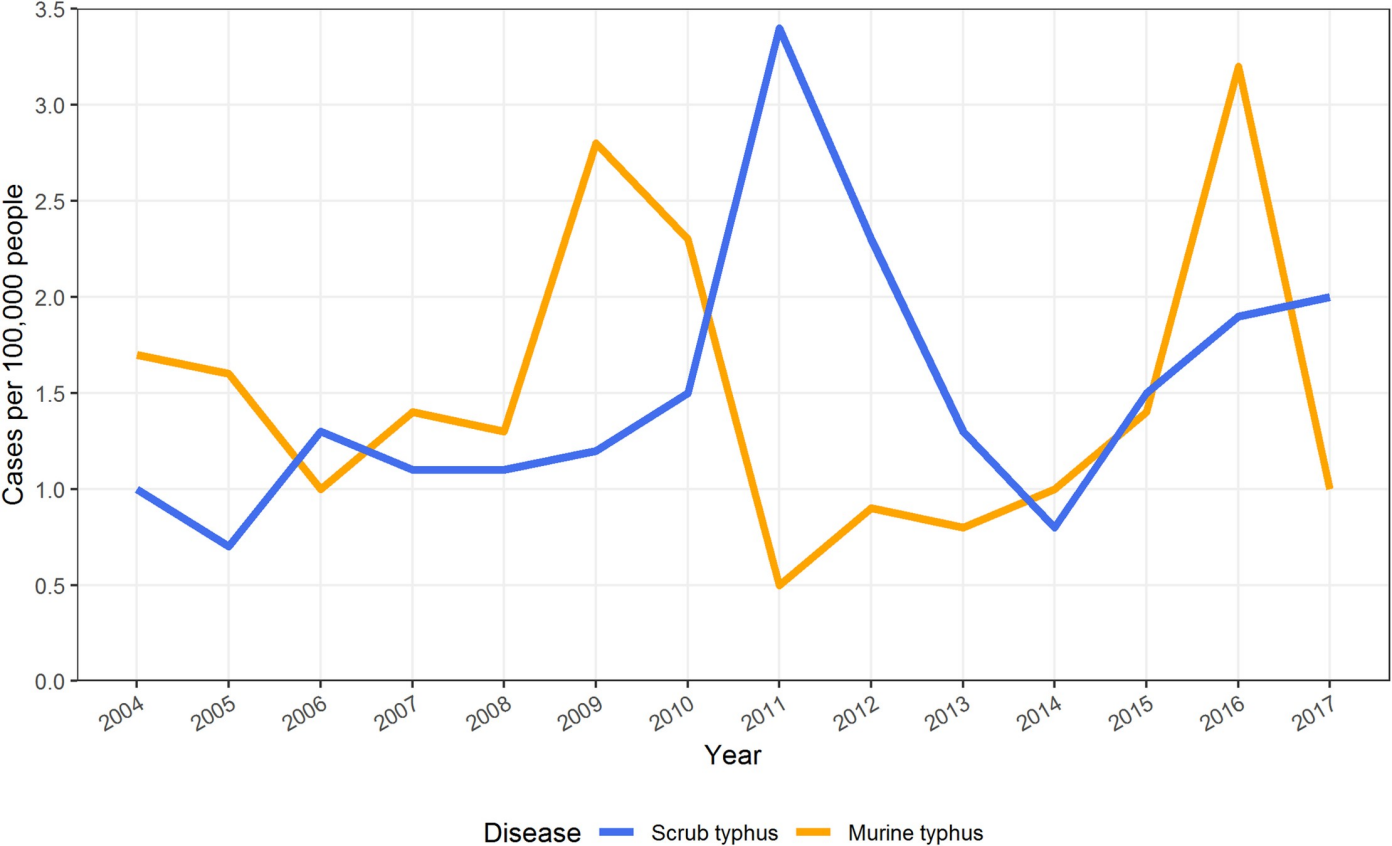
Yearly incidence of scrub typhus and murine typhus in Laos between 2004 and 2017.

**Fig 4:**
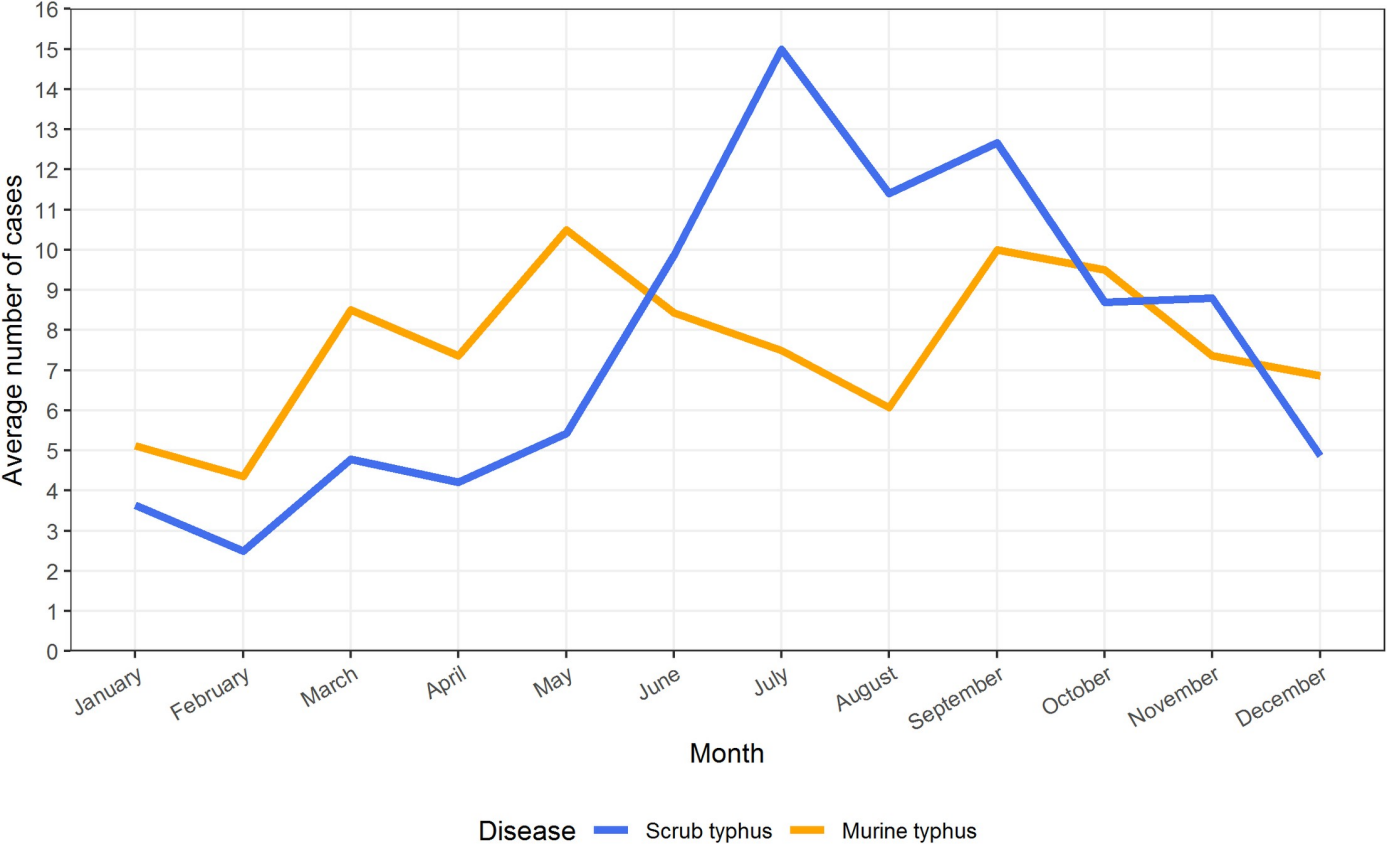
Mean number of patients with scrub typhus and murine typhus per month in Laos between 2004 and 2017.

**Fig 5:**
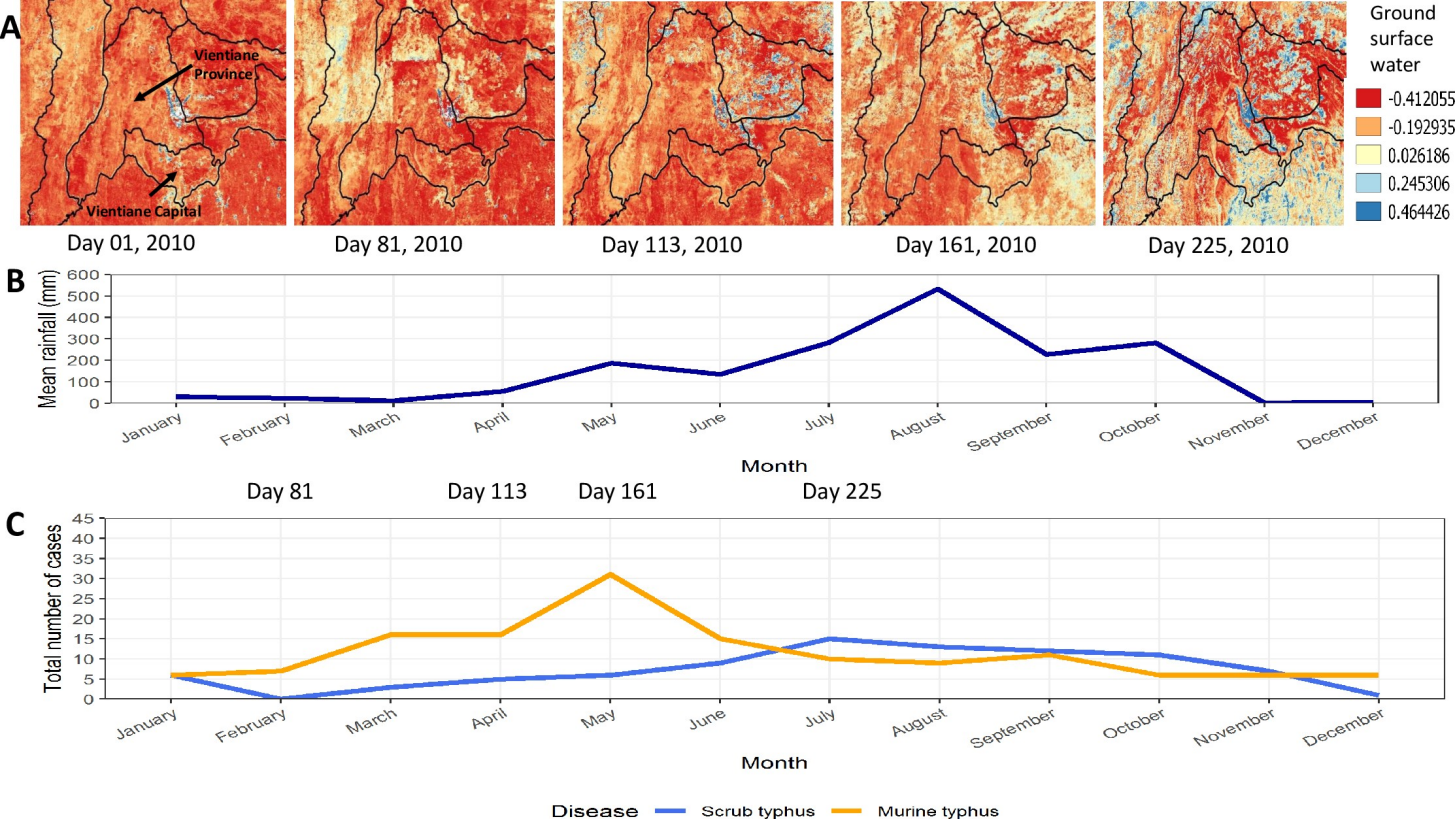
Year 2010. A. Amount of surface water for Vientiane Capital and Vientiane Province B. Combined mean rainfall per month for Vientiane Capital and Vientiane Province C. Number of patients with scrub typhus and murine typhus for Vientiane Capital and Vientiane Province per month. Maps were created using QGIS version 3.4.9. All layers were created by the authors of this manuscript.

**Fig 6:**
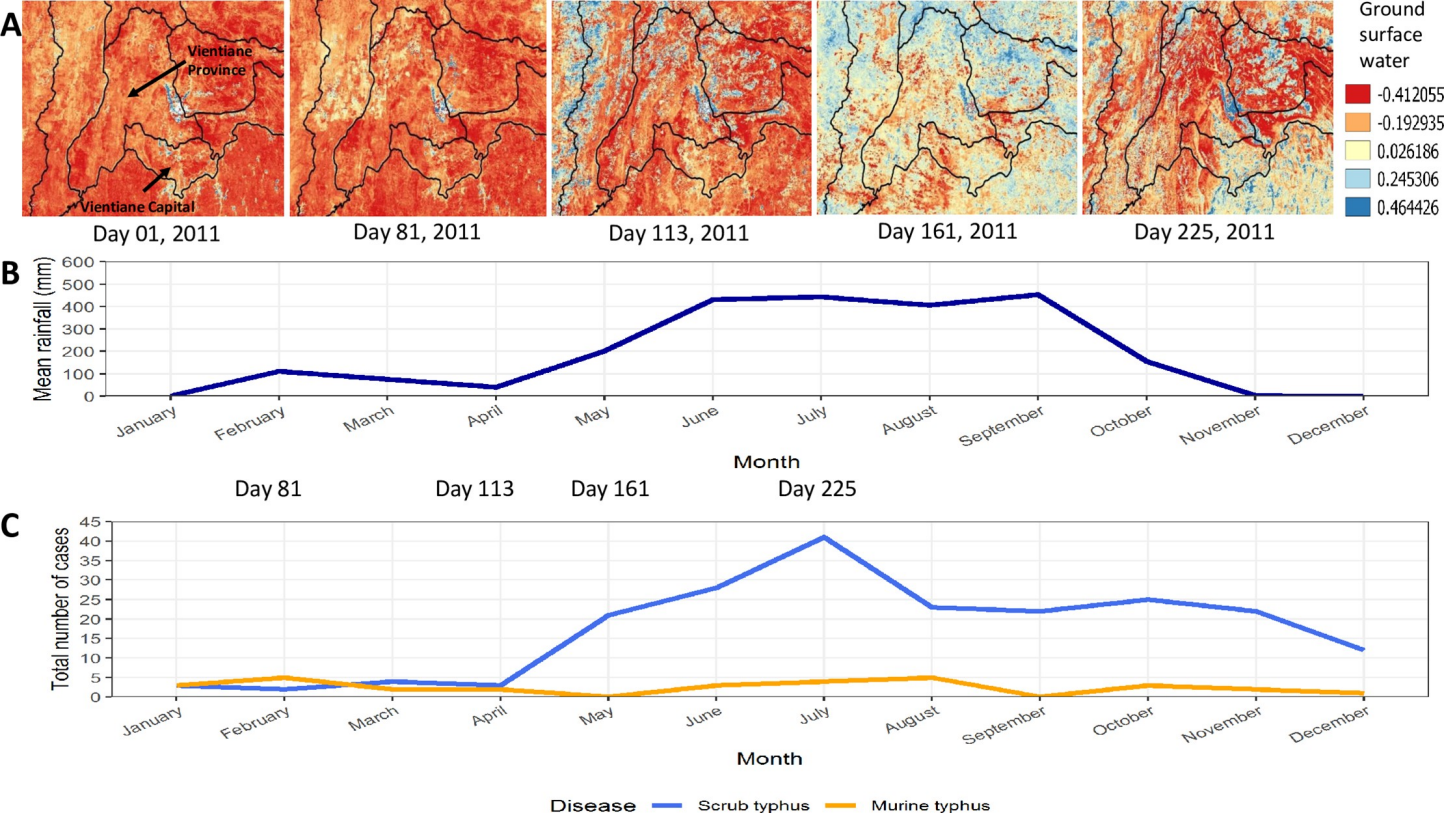
Year 2011. A. Amount of surface water for Vientiane Capital and Vientiane Province B. Combined mean rainfall per month for Vientiane Capital and Vientiane Province C. Number of patients with scrub typhus and murine typhus from Vientiane Capital and Vientiane Province per month. Maps were created using QGIS version 3.4.9. All layers were created by the authors of this manuscript.

Years of peak MT incidence corresponded to years with more provinces reporting an incidence > 0.1 per 100,000 (2009 and 2016 in Figs 2 and 3). MT tended to peak in the dry months of April/ May while ST peaked in the wetter months of July/August (Figs 4–6).

### Meteorological and environmental analyses

Rainfall or associated seasonal climatic factors had a differential impact on the epidemiology of both diseases. The total precipitation and mean temperature from Vientiane Capital over the study period are shown in S2 Fig. In the univariable regression analyses, ST hospital incidence for patients from Vientiane Capital was positively associated with higher minimum temperature, humidity, precipitation, number of rainy days and number of days with thunderstorms (Table 3). These associations remained robust when examining ST patients from all Lao provinces (in relation to climatic data from Vientiane Capital). MT hospital incidence for patients from Vientiane Capital was positively associated with higher temperature and maximum sustained wind speed (Table 3). Univariable analysis including all MT patients from all provinces (using Vientiane Capital climate data), observed similar associations and additionally identified a positive association with the number of days with thunderstorms (Table 4). At the countrywide level, ST hospital incidence was positively associated with humidity in the multivariable model (Table 5). MT hospital incidence was positively associated with mean temperature in the multivariable models (Table 6).

**Table 3:** Univariable regression analysis of climatic factors associated with scrub typhus and murine typhus from patients in Vientiane Capital, using climate data from Vientiane Capital.

Climate factor	Scrub typhus (841 patients)			Murine typhus (989 patients)		
	Estimate	Standard error	p value	Estimate	Standard error	p value
Mean temperature (°C)	0.094	0.048	0.051	**0.084**	**0.029**	**0.004**
Maximum temperature (°C)	0.022	0.051	0.662	**0.092**	**0.032**	**0.003**
Minimum temperature (°C)	**0.111**	**0.039**	**0.004**	**0.050**	**0.023**	**0.030**
Mean relative humidity (%)	**0.056**	**0.013**	**<0.001**	-0.001	0.009	0.989
Total rainfall (mm)	**0.003**	**0.001**	**<0.001**	0.003	0.000	0.495
Mean visibility (Km)	0.104	0.071	0.146	0.050	0.046	0.280
Mean wind speed (Km/h)	0.088	0.191	0.647	0.113	0.120	0.347
Maximum sustained wind speed (Km/h)	0.108	0.057	0.058	**0.090**	**0.036**	**0.012**
Total days rained	**0.048**	**0.011**	**<0.001**	0.010	0.007	0.179
Total days with thunderstorm	**0.046**	**0.017**	**0.006**	0.020	0.011	0.069
Total days with fog	-0.105	0.275	0.703	-0.110	0.172	0.520

**Table 4:** Univariable regression analysis of climatic factors associated with scrub typhus and murine typhus from country wide case counts using climate data from Vientiane Capital.

Climate factor	Scrub typhus (1,286 patients)			Murine typhus (1,285 patients)		
	Estimate	Standard error	p value	Estimate	Standard error	p value
Mean temperature (°C)	**0.111**	**0.036**	**0.002**	**0.088**	**0.028**	**0.002**
Maximum temperature (°C)	0.010	0.040	0.796	**0.096**	**0.031**	**0.002**
Minimum temperature (°C)	**0.129**	**0.028**	**<0.001**	**0.054**	**0.023**	**0.016**
Mean relative humidity (%)	**0.069**	**0.010**	**<0.001**	0.001	0.009	0.873
Total rainfall (mm)	**0.003**	**0.001**	**<0.001**	0.000	0.000	0.689
Mean visibility (Km)	**0.129**	**0.057**	**0.023**	0.068	0.045	0.132
Mean wind speed (Km/h)	-0.182	0.151	0.226	0.157	0.118	0.183
Maximum sustained wind speed (Km/h)	0.031	0.046	0.503	**0.102**	**0.035**	**0.004**
Total days rained	**0.047**	**0.008**	**<0.001**	0.012	0.007	0.108
Total days with thunderstorm	**0.046**	**0.013**	**<0.001**	**0.023**	**0.011**	**0.032**
Total days with fog	-0.079	0.211	0.707	-0.065	0.167	0.695

**Table 5:** Multivariable regression analysis of climatic factors associated with scrub typhus from country wide case counts, using climate data from Vientiane Capital (1,286 patients). Model includes average temperature, average relative humidity and total rainfall.

Climate factor	Estimate	Standard error	p value
Mean temperature (°C)	0.005	0.035	0.894
Mean relative humidity (%)	**0.067**	**0.017**	**<0.001**
Total rainfall (mm)	0.000	0.001	0.917

**Table 6:** Multivariable regression analysis of climatic factors associated with murine typhus from country wide case counts, using climate data from Vientiane Capital. Model includes mean temperature and maximum sustained wind speed (1,285 patients).

Climate factor	Estimate	Standard error	p value
Mean temperature (°C)	**0.064**	**0.031**	**0.039**
Maximum sustained wind speed (Km/h)	0.063	0.038	0.101

The univariate analysis of environmental and geographic variables indicated that people who were diagnosed with ST were from villages that were farther from major roads than those diagnosed with MT; higher in elevation than those with MT; from villages with smaller populations than those with MT; from villages with higher variation in NDFI and EVI; and from villages that recently had higher NDFI and EVI (when compared to home villages of MT patients or patients without a typhus diagnosis). Conversely, people who were diagnosed with MT were from villages that were close to major roads, higher in population size and lower in elevation. Mean NDFI was higher in home villages of MT patients when compared to those of ST patients and those of patients who did not receive a typhus diagnosis (S3 Fig).

**Fig 7:**
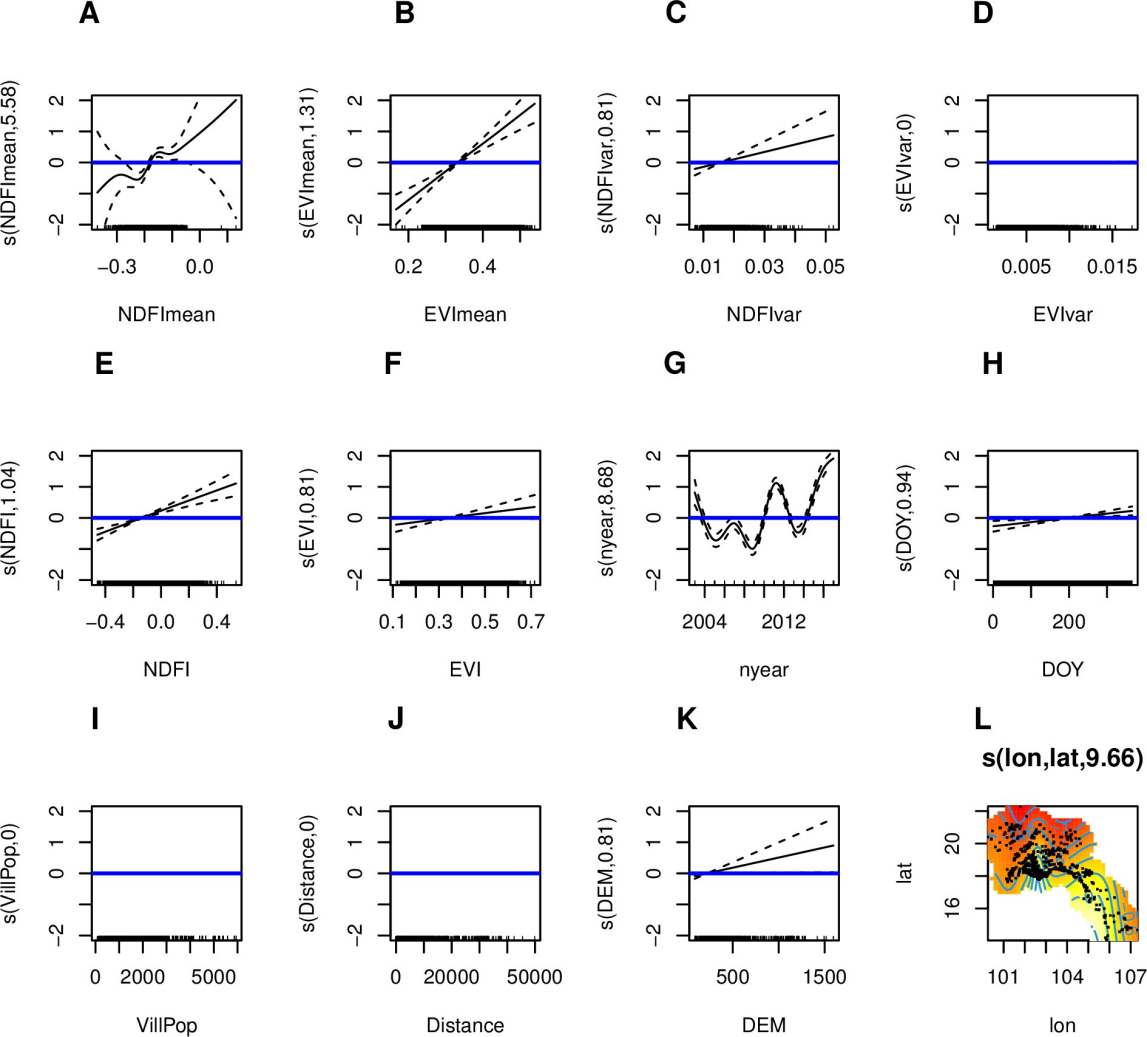
Results from generalized additive logistic regression for the geographic, demographic and environmental predictors of scrub typhus infection. A.) mean NDFI (normalized difference flooding index) for patient home village across study period; B.) mean EVI (enhanced vegetation index) for patient home village across study period; C.) variance in NDFI for patient home village across study period; D.) variance in EVI for patient home village across study period; E.) NDFI around patient home village in 16 days leading up to hospital admission; F.) EVI around patient home village in 16 days leading up to hospital admission; G.) year of hospital admission; H.) day of year of hospital admission; I.) population of patient home village; J.) distance from patient home village to nearest major road; K.) elevation of patient home village; L.) geographic coordinates of patient home village. Plots above the blue line (at the y-intercept) indicate a positive association for a given covariate, for the given x-axis value. For the geographic coordinates (panel L), lighter colored areas on the map indicate places with higher baseline risk of being diagnosed with scrub typhus. P-values are indicated in S5 Table.

**Fig 8:**
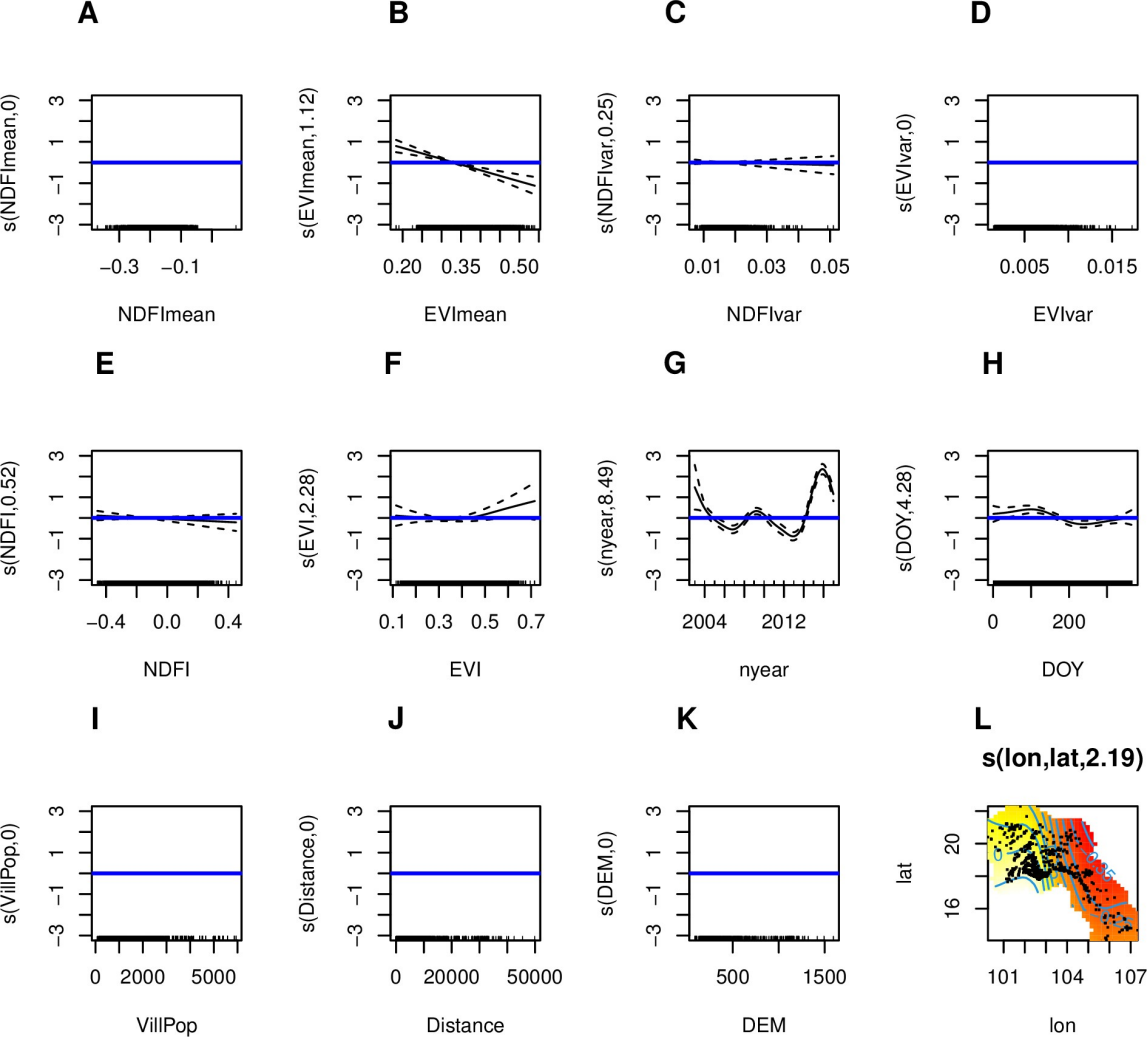
Results from generalized additive logistic regression for the geographic, demographic and environmental predictors of murine typhus infection. A.) mean NDFI (normalized difference flooding index) for patient home village across study period; B.) mean EVI (enhanced vegetation index) for patient home village across study period; C.) variance in NDFI for patient home village across study period; D.) variance in EVI for patient home village across study period; E.) NDFI around patient home village in 16 days leading up to hospital admission; F.) EVI around patient home village in 16 days leading up to hospital admission; G.) year of hospital admission; H.) day of year of hospital admission; I.) population of patient home village; J.) distance from patient home village to nearest major road; K.) elevation of patient home village; L.) geographic coordinates of patient home village. Plots above the blue line (at the y-intercept) indicate a positive association for a given covariate, for the given x-axis value. For the geographic coordinates (panel L), lighter colored areas on the map indicate places with higher baseline risk of being diagnosed with murine typhus. P-values are indicated in S5 Table.

From the generalized additive logistic regressions, most ST diagnoses occurred in the later part of the annual rainy season, July-September (Fig 7H). Patients with ST infection were more likely to come from villages with higher levels of surface flooding and vegetation in the 16 days before diagnosis (Fig 7E and 7F, S5 Table), and villages with higher mean surface flooding and vegetation in general (Fig 7A and 7B, S5 Table). Furthermore, patients who were diagnosed with ST were more likely to come from villages at higher elevations (Fig 7K, S5 Table). ST patients were dispersed throughout Vientiane Capital and Vientiane Province, whereas MT cases occurred closer to the city centre and along the Mekong River (Figs 7L and 8L).

## Discussion

Scrub typhus and murine typhus are common causes of febrile illness in Laos. Approximately 17% of inpatients suspected of typhus infection tested positive for either disease. The geographical range of provinces from which ST patients arose increased throughout the study period (Fig 1). This may reflect expanded surveillance, diagnostic testing and research studies, enhanced awareness of the disease and changing landscapes and climate.

Though ST has been considered a rural disease, it has been described from urban areas including in Vietnam [14,15], South Korea [16] and China [17]. Regarding ST as a purely rural disease may mislead clinicians and should be considered in the differential for patients with fever from both urban and rural areas. A serosurvey of ST and MT IgG in Vientiane City found that ST exposure was more common in peri-urban areas on the city outskirts and MT was more common in the more urban city central areas, however there was significant overlap [6]. Our data, which extends beyond the previous study locations, are consistent with these findings. However, unlike some other Asian capitals, Vientiane City has significant agricultural land within the city, and many urban inhabitants have unusually high exposure to nearby rural habitats. Puzzlingly, data from south India and Bangladesh show less urban-rural disparity between the incidence of the two diseases [18,19]. The majority of patients came from Vientiane City, which is the main catchment area for the hospital. During the 14-year span of this analysis, Vientiane Capital’s built up area moved centrifugally to the north, north-east and north-west of the centre. This spread of urbanized landscapes across rural areas (including rice paddies and forests) may lead to more people coming into contact with vectors of both diseases. We therefore hypothesised that the more urban disease MT would have spread outwards whilst the more rural ST would have progressively reduced incidence nearer the centre of the city; the data are consistent with this hypothesis and suggests unrecognised health risks of expanding urbanisation (see S1 Document).

Most patients came from villages located along major roads (S1 Fig). This probably reflects that patients living near roads have better access to healthcare facilities and therefore are more likely to be tested and treated. However, it could also mean that the small mammals that carry chiggers and fleas are more populous astride these main roads due to markets heightening the risk of exposure. The ranging patterns and behaviour of people, chiggers, fleas and rodents in Laos and the ecology of ST and MT have been little studied, but doing so may identify remediable risk factors.

Though location information was available for patients’ villages, with an incubation period of 6–20 days for ST and 6–14 days for MT [20,21], it is difficult to know where disease acquisition occurred. Visiting a forest or a rice field two weeks prior to symptom onset was positively associated with ST infection but negatively associated with MT, consistent with the evidence that exposure to rural habitats increases the risk of ST. In Vietnam occupational contact with farming and forest environments were associated with ST infection but not MT infection [15]. In Thailand and China agricultural workers made up the largest percentage of ST patients [20,22]. There also appears to be a significant relationship between ST risk and surface flooding and vegetation. Villages with higher levels of surface water and vegetation tended to be in rural settings with flooded rice fields. This relationship with water has been recognized in Japan, Korea and, in WW2, on the India/Burma border [23,24].

ST incidence peaked in the wet months of June to September whereas MT peaked in the drier months of April to May (Figs 4 and S2). These contrasting seasonal patterns of MT and ST have been clinically apparent at Mahosot Hospital since 2002. In and around Vientiane Capital ST appears to have a much stronger association with seasonal climatic variables than MT. ST was positively associated with higher temperature, humidity, precipitation and reduced visibility in the univariable analyses, and was associated with higher humidity in the multivariable analysis. Similar results were seen in northern and north-eastern Thailand with ST cases associated with monthly rainfall and average monthly temperature [20]. MT incidence was associated with higher temperature and maximum wind speed in the univariable analysis, but only weakly associated with higher temperature in the multivariable analysis. The two diseases seem to show different cycles, with MT incidence tending to follow El Niño, when hot temperatures and drought occurred in South-East Asia at the end of 2009, start of 2010 and 2015/2016 when MT cases peaked whereas ST follows the wet years of La Niña as seen in 2011 and end of 2016 and 2017 [25,26]. In Guangzhou, China, a 10% increase in humidity was associated with an 8.5% increased odds for ST infection with a 4-month lag but showed a 5-month lag after an El Niño event [27].

Little is known about the distribution of chigger vectors and their hosts in Laos. Both higher temperature and rainfall are important determinants of chigger population numbers [23]. Chigger survival after submersion under water for two weeks has been recorded, with potential relevance for flooded rice fields [23,28]. ST temporal patterns differ between and even within countries, especially across latitudes, with temperature important further north where human disease is more confined to a specific season. Closer to the equator rainfall may be more important and there is less seasonal fluctuation in case numbers [20,23].

There is also little known about flea distribution on animals and across Laos. In Vietnam, fleas were found on rodents closer to inhabited areas and flea numbers declined the further away rodents were from villages [29]. The life cycle of fleas lasts weeks to months depending on environmental conditions, with ideal conditions being higher temperature and humidity [21]. Flea eggs hatch in warmer months, resulting in higher numbers of fleas and more chances of contact with people during the dry, hot months [29]. This could partially explain the peak in MT cases in the dry months of April/May and why MT is found predominantly in urban areas where fleas are less affected by the rain. Plague (*Yersinia pestis*), also transmitted by fleas, may have a similar disease ecology to MT [30,31]. Analysis of climate suggests that the risk of human plague infection falls when monthly rainfall is <10mm [31,32]. Determining whether such relationships exist for MT could help disease prediction.

Weather patterns impact the natural cycle of vectors as well as the behavior of humans. Rice farming is a major component of the Lao economy; planting rice generally occurs at the start of the wet season in May/June with harvesting in October/November. Interestingly, these are the peak months for ST cases, suggesting that increased intensity of agricultural work raises the risk of contracting ST. Chigger numbers may also be higher during wetter seasons. With climatic factors and visiting a rice field associated with ST infection, it remains uncertain whether these factors are independently associated, act in concert or are confounders (S4 Fig).

There are several limitations to this study. All ST and MT diagnoses were based on antibody-detecting RDTs alone which have reduced sensitivity and specificity in comparison to the reference standard IFA and PCR: RDTs have sensitivity and specificity ranging from 23–100% and 73–100%, respectively [9,33,34]. In Laos, RDTs are convenient because they do not require significant staff levels of training and results are available within an hour of the sample being obtained. There was a stock-out of ST RDTs from September 2015 to March 2016 and different ST RDTs were used and this could have influenced our findings. These data arise from inpatient samples and therefore may underestimate the community burden. Health seeking behaviour and access to healthcare will have key impacts on who presents to hospital where and when. Due to variable climate data availability and quality among Lao provinces, all meteorological analyses relied on data from Vientiane Capital, limiting the resolution of meteorological analyses. There are substantial missing values for the univariable and multivariable analysis which may have resulted in bias towards certain results. Unfortunately we were unable to find the missing information due to the length of time of the study and lack of follow-up for patients. Discharge status data was also not recorded and would have been valuable to add further information about the mortality rates of the diseases in Laos.

Conversely, a major strength of this analysis lies in the long-term (14 years) collection of detailed clinical records for both ST and MT, in a region that is endemic for both diseases. In this research we found that there are temporal trends that extend beyond seasons. We also found that patients diagnosed with ST were more likely to come from villages with higher amounts of surface flooding and higher vegetation density (which is also related to precipitation and surface water), regardless of season or year. Further research on the ecology and epidemiology of both diseases is needed and these results can aid clinicians who are commonly faced with illnesses of unknown aetiology.

In areas with sympatric ST and MT these data suggest that residence, occupational and climatic differential risks are useful for identifying patients. Importantly, as the data suggest that as cities expand high risk areas for MT will expand, we need to be prepared to mitigate this [35]. With global heating and risks of attendant higher precipitation, these data suggest that both the incidence and spatial distribution of MT and ST will increase. With the known distribution of scrub typhus greatly expanded over the last few years, it is clear that disease burden is underestimated and global heating may further increase both distribution and incidence of these diseases; more surveillance and multidisciplinary research is needed.

Further research is needed to understand the mechanisms of environmental/climatic influences on their epidemiology, especially for flooding and ST, to inform interventions. Both diseases are severely neglected and much more investment is needed to improve the current woeful situation for accessible and accurate diagnostic tests to understand their global epidemiology, understand disease drivers and diagnose and treat individual patients.

## Supporting information

S1 Document Details of spatio-temporal analyses. (DOCX)

S1 Table Variables and descriptions from the generalized additive logistic regressions. (DOCX)

S2 Table Univariable and multivariable logistic regression for factors associated with scrub typhus positive patients from all patients tested for scrub typhus by RDT living in Vientiane Capital (878 scrub typhus patients from 5,533 patients tested). n = number of patients with feature, N = number of patients with result (%). The data from all individual patients were included in the study although denominators vary for different parameters due to incomplete data.
(DOCX)

S3 Table Linear regression results for distance to Vientiane City (in kilometres). (DOCX)

S4 Table Linear regression results for distance to major road (in meters). (DOCX)

S5 Table Model output from generalized additive logistic regressions for both scrub typhus and murine typhus. Interpretation of coefficients is best by visualizing the plots of smoothed effects (Figs 7 and 8). Results from univariate analyses are presented in S3 Fig. Variable names correspond to S1 Table.
(DOCX)

S1 Fig Map of roads and villages of patients with scrub typhus and murine typhus in Vientiane Capital and Vientiane Province. A. Scrub typhus patient villages in Vientiane Capital and Vientiane Province B. Murine typhus patient villages in Vientiane Capital and Vientiane Province.
(TIF)

S2 Fig Total precipitation (mm) and the mean temperature (°C) for Vientiane Capital 2004–2015. (TIF)

S3 Fig Summary statistics for environmental and geographic indices of patient home villages, by diagnosis. The data are split into those who were diagnosed as having scrub typhus (“ST”); murine typhus (“MT”); both ST and MT (“Mixed”); or “Neither” (indicating patients who were suspected of having typhus but were not diagnosed with either ST or MT).
(TIF)

S4 Fig Epidemiological triad for both murine typhus and scrub typhus. Environmental components are derived from this analysis, and some have been previously reported. We hypothesize that these environmental factors act on both vector and human populations, that humans also change the environment, and that these combinations of interactions lead to increased contact between vectors and susceptible hosts in different geographic locations and at different times (e.g. hot season for murine typhus and rainy season for scrub typhus).
(TIF)
