# Supplementary material for: A spatio-temporal analysis of scrub typhus and murine typhus in Laos; implications from changing landscapes and climate
Source: PLoS Negl Trop Dis. 2021 Aug 25;15(8):e0009685. doi: 10.1371/journal.pntd.0009685 (PMC8386877; doi:10.1371/journal.pntd.0009685)
Supplement: S1 Document — (DOCX) [file pntd.0009685.s001.docx]

**S1 Document**. Details of spatio-temporal analyses

Environmental indices (NDFI, NDVI, and EVI)

The photosynthetic components of vegetation (i.e. chlorophyll) absorb visible light, especially Red and Blue wavelengths. Most infrared radiation is reflected by healthy vegetation and the contrast between Red and near-infrared (NIR) reflection therefore provides an estimate of healthy vegetation.

A commonly used measure of landscape vegetation is the normalized difference vegetation index (NDVI [1]) which is specified as:

$$NDVI= \frac{NIR-Red}{NIR+Red}$$

This indice is however sensitive to atmospheric effects and dense canopy structure [2]. In areas with high vegetation density NDVI quickly becomes saturated. An improved metric has been developed to account for these problems, commonly referred to as the enhanced vegetation index (EVI [3]). This metric uses the difference between Red and Blue reflectances as an estimator of atmospheric influence level on the vegetation index. EVI is specified as:

$$EVI=G\frac{NIR-Red}{NIR+C_{1}Red-C_{2}Blue+L}$$

; where *L* is the canopy background adjustment;

*C*_1_ and *C*_2_ are coefficients of an aerosol resistance term;

and *G* is a scaling factor.

A variety of indices have also been proposed to measure surface water or water content within vegetation (i.e. measuring drought conditions or identifying areas that have been burned). In general, indices that use a combination of visible spectral regions (VIS) and SWIR (short wave infra-red) are usually proposed for identifying water bodies. Infrared in SWIR wavelengths are well- absorbed by water (see [4], for example). Following Boschetti et al. [5] we use the following normalized flooding index (NDFI):

$$NDFI=\frac{Red-SWIR2}{Red+SWIR2}$$

; where SWIR2 is shortwave infrared radiation 2 (~ 1640nm).

Analysis of the distance from patient home village to: A.) Vientiane Capital (where Mahosot Hospital is located) over the study period, and B.) the nearest major road.

Mahosot Hospital is located in Vientiane City. We hypothesized that over time, patients diagnosed with either scrub typhus or murine typhus would be coming from farther away from the capital, as diagnostic capabilities spread from Mahosot Hospital into surrounding rural areas and as the urban environment of Vientiane spread into the countryside. In order to test this hypothesis, we calculated the distance from all patient home villages to the Phatuxay monument (lat: 17.9706; lon: 102.619) in central Vientiane City. We then used a linear regression to test for increasing distance to Vientiane City over time. The output of the linear regression was the linear distance to Phatuxay Monument and predictor variables (covariates) were the year of diagnosis and the diagnosis outcome (ST, MT, mixed, or neither).

The distance from Vientiane City increased over the study period. For each one year increase there was a corresponding 0.85km increase in distance of suspected typhus patients from Vientiane City. ST patients came from farther away (mean of 19.2km) than MT patients.

We used the road networks from OpenStreetMaps (<http://www.openstreetmap.la/>) to measures the straight line distance between all patient villages and the nearest major road. Major roads were selected by taking “primary”, “secondary”, and all major connecting roads from OpenStreetMaps Laos (downloaded in February 2017). Primary roads are paved roads that link major towns and cities and secondary roads are paved roads that link mid-sized towns. Link roads for both are ramps that connect other roads to primary or secondary roads.

The distance from major roads increased over the study period. For each one year increase there was a corresponding 67.9 meter increase in distance from a major road. ST patients came from almost 1km (993.7 meters) away from major roads when compared to MT patients.

References

1. Rouse J, Hass R, Deering D, Sehell J. Monitoring the vernal advancement and retrogradation (Green wave effect) of natural vegetation [Internet]. 1974 [cited 2019 Jul 29] p. 8. Report No.: E74-10676, NASA-CR-139243, PR-7. Available from: <https://ntrs.nasa.gov/archive/nasa/casi.ntrs.nasa.gov/19740022555.pdf>

2. Huete AR. A soil-adjusted vegetation index (SAVI). Remote Sensing of Environment. 1988 Aug 1;25(3):295–309.

3. Huete A, Didan K, Miura T, Rodriguez E, Gao X, Ferreira L. Overview of the radiometric and biophysical performance of the MODIS vegetation indices. Remote Sensing of Environment. 2002;83:195–213.

4. Hale GM, Querry MR. Optical constants of water in the 200-nm to 200-μm wavelength region. Applied optics. 1973;12(3):555-63.

5. Boschetti M, Nutini F, Manfron G, Brivio PA, Nelson A. Comparative analysis of normalised difference spectral indices derived from MODIS for detecting surface water in flooded rice cropping systems. PloS one. 2014;9(2):e88741. doi: 10.1371/journal.pone.0088741. PubMed PMID: 24586381; PubMed Central PMCID: PMCPMC3930609.
