# Supplementary material for: A spatio-temporal analysis of scrub typhus and murine typhus in Laos; implications from changing landscapes and climate
Source: PLoS Negl Trop Dis. 2021 Aug 25;15(8):e0009685. doi: 10.1371/journal.pntd.0009685 (PMC8386877; doi:10.1371/journal.pntd.0009685)
Supplement: S1 Table — (DOCX) [file pntd.0009685.s002.docx]

**S1 Table.** Variables and descriptions from the generalized additive logistic regressions

| **Variable** | **Description** |
| --- | --- |
| NDFImean | mean flooding index for patient home village |
| EVImean | mean vegetation index for patient home village |
| NDFIvar | variance of flooding index for patient home village |
| EVIvar | variance of vegetation index for patient home village |
| EVI | time varying vegetation index for patient home village (in the previous 16 days) |
| NDFI | time varying flooding index for patient home village (in the previous 16 days) |
| nyear | year |
| DOY | day of year |
| VillPop | population estimate from patient home village |
| Distance | distance from patient home village to nearest major road |
| DEM | estimated elevation of patient home village |
| lat/lon | geographic coordinates of patient home village |
