# Supplementary material for: A spatio-temporal analysis of scrub typhus and murine typhus in Laos; implications from changing landscapes and climate
Source: PLoS Negl Trop Dis. 2021 Aug 25;15(8):e0009685. doi: 10.1371/journal.pntd.0009685 (PMC8386877; doi:10.1371/journal.pntd.0009685)
Supplement: S2 Table — n = number of patients with feature, N = number of patients with result (%). The data from all individual patients were included in the study although denominators vary for different parameters due to incomplete data. (DOCX) [file pntd.0009685.s003.docx]

**S2 Table**. Univariable and multivariable logistic regression for factors associated with scrub typhus positive patients from all patients tested for scrub typhus by RDT living in Vientiane Capital (878 scrub typhus patients from 5,533 patients tested). n= number of patients with feature, N= number of patients with result (%). The data from all individual patients were included in the study although denominators vary for different parameters due to incomplete data.

| **Factor** | **Admission features for all patients tested for scrub typhus**  n/N (%) | **Admission features of scrub typhus positive patients**  n/N (%) | **Univariable analysis** | | | **Multivariable analysis** | | |
| --- | --- | --- | --- | --- | --- | --- | --- | --- |
|  |  |  | **OR** | **95% CI** | **p-value** | **OR** | **95% CI** | **p-value** |
| Visited a rice field in last 2 weeks | 298/891 (33) | 180/387 (47) | **2.84** | **2.13- 3.79** | **<0.001** | **1.54** | **1.08.- 2.20** | **0.017** |
| Visited a forest in last 2 weeks | 246/867 (28) | 169/371 (46) | **4.55** | **3.31- 6.25** | **<0.001** | **3.38** | **2.33- 4.89** | **<0.001** |
| Seen rat in last 2 weeks | 2,349/2,562 (92) | 442/528 (84) | **0.34** | **0.26- 0.46** | **<0.001** | 0.81 | 0.56- 1.18 | 0.275 |
| Cat at home | 1,500/2,826 (53) | 285/551 (52) | 0.93 | 0.77- 1.13 | 0.478 | **0.64** | **0.47- 0.88** | **0.005** |
