# Supplementary material for: A spatio-temporal analysis of scrub typhus and murine typhus in Laos; implications from changing landscapes and climate
Source: PLoS Negl Trop Dis. 2021 Aug 25;15(8):e0009685. doi: 10.1371/journal.pntd.0009685 (PMC8386877; doi:10.1371/journal.pntd.0009685)
Supplement: S4 Table — (DOCX) [file pntd.0009685.s005.docx]

**S4 Table.** Linear regression results for distance to major road (in meters)

| **Covariate** | **estimate** | **SE** | **t-value** | **p-value** | **count** |
| --- | --- | --- | --- | --- | --- |
| *Year of diagnosis* | 67.88 | 19.96 | 3.40 | 0.001 | 5,757 |
| *MT diagnosis* | Comparison |  |  |  | 1,125 |
| *Neither MT nor ST* | 777.63 | 186.77 | 4.16 | <0.001 | 3,384 |
| *ST diagnosis* | 993.68 | 223.95 | 4.44 | <0.001 | 1,173 |
| *Mixed infection* | -538.57 | 640.03 | -0.84 | 0.400 | 75 |

*A total of 5,757 patients with complete data were included in this analysis. Two rows of data were dropped because of missing admission dates (both patients with neither ST nor MT positive).
