# Supplementary material for: A spatio-temporal analysis of scrub typhus and murine typhus in Laos; implications from changing landscapes and climate
Source: PLoS Negl Trop Dis. 2021 Aug 25;15(8):e0009685. doi: 10.1371/journal.pntd.0009685 (PMC8386877; doi:10.1371/journal.pntd.0009685)
Supplement: S5 Table — Interpretation of coefficients is best by visualizing the plots of smoothed effects (Figs 7 and 8). Results from univariate analyses are presented in S3 Fig. Variable names correspond to S1 Table. (DOCX) [file pntd.0009685.s006.docx]

**S5 Table.** Model output from generalized additive logistic regressions for both scrub typhus and murine typhus. Interpretation of coefficients is best by visualizing the plots of smoothed effects (Figs 7 and 8). Results from univariate analyses are presented in S2 Fig. Variable names correspond to S1 Table. A total of 5,433 patients with complete data were included in the model for scrub typhus and a total of 5,100 patients with complete data were included in the model for murine typhus. Only patients with complete data for all variables were included in each respective model.

|  | Scrub typhus | | | | Murine typhus | | | |
| --- | --- | --- | --- | --- | --- | --- | --- | --- |
|  | EDF | Ref.df | Chi.sq | p-value | EDF | Ref.df | Chi.sq | p-value |
| **s(NDFImean)** | **5.58** | **9** | **34.61** | **<0.001** | 0.00 | 9 | 0.00 | 0.351 |
| **s(EVImean)** | **1.31** | **9** | **48.27** | **<0.001** | **1.12** | **9** | **33.58** | **<0.001** |
| **s(NDFIvar)** | **0.81** | **9** | **3.99** | **0.020** | 0.25 | 9 | 0.34 | 0.237 |
| s(EVIvar) | 0.00 | 9 | 0.00 | 0.543 | 0.00 | 9 | 0.00 | 0.696 |
| **s(EVI)** | **0.81** | **9** | **3.63** | **0.029** | **2.28** | **9** | **8.27** | **0.007** |
| **s(NDFI)** | **1.04** | **9** | **41.12** | **<0.001** | 0.52 | 9 | 1.07 | 0.117 |
| **s(nyear)** | **8.68** | **9** | **517.98** | **<0.001** | **8.49** | **9** | **476.10** | **<0.001** |
| **s(DOY)** | **0.94** | **9** | **10.64** | **<0.001** | **4.28** | **9** | **31.94** | **<0.001** |
| s(VillPop) | 0.00 | 9 | 0.00 | 0.618 | 0.00 | 9 | 0.00 | 0.521 |
| s(Distance) | 0.00 | 9 | 0.00 | 0.979 | 0.00 | 9 | 0.00 | 0.811 |
| **s(DEM)** | **0.81** | **9** | **4.34** | **0.010** | 0.00 | 9 | 0.00 | 0.342 |
| **s(lon,lat)** | **9.66** | **49** | **34.37** | **<0.001** | **2.19** | **49** | **5.08** | **0.048** |
