# Supplementary figures and images for: A spatio-temporal analysis of scrub typhus and murine typhus in Laos; implications from changing landscapes and climate
Source: PLoS Negl Trop Dis. 2021 Aug 25;15(8):e0009685. doi: 10.1371/journal.pntd.0009685 (PMC8386877; doi:10.1371/journal.pntd.0009685)

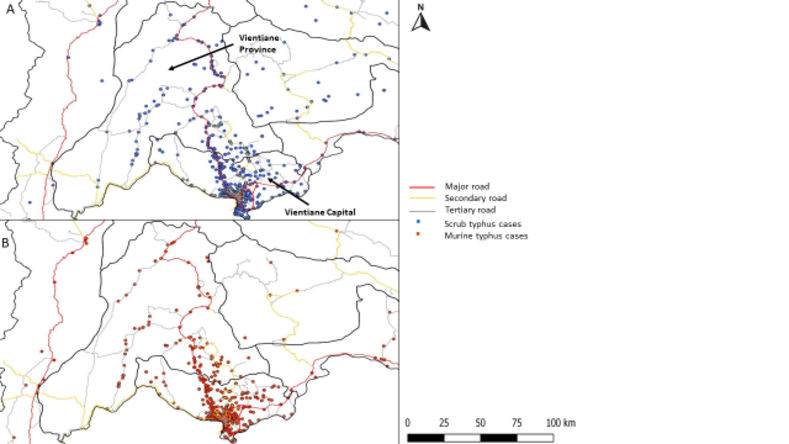

Supplement: S1 Fig — A. Scrub typhus patient villages in Vientiane Capital and Vientiane Province B. Murine typhus patient villages in Vientiane Capital and Vientiane Province. (TIF) [file pntd.0009685.s007.tif]

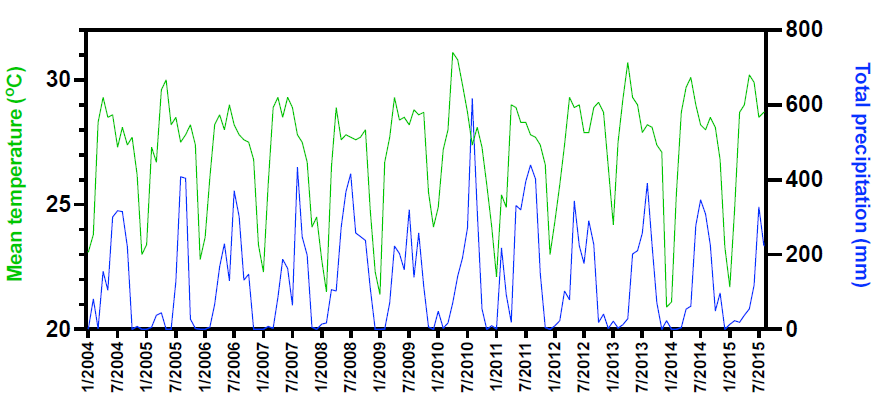

Supplement: S2 Fig — (TIF) [file pntd.0009685.s008.tif]

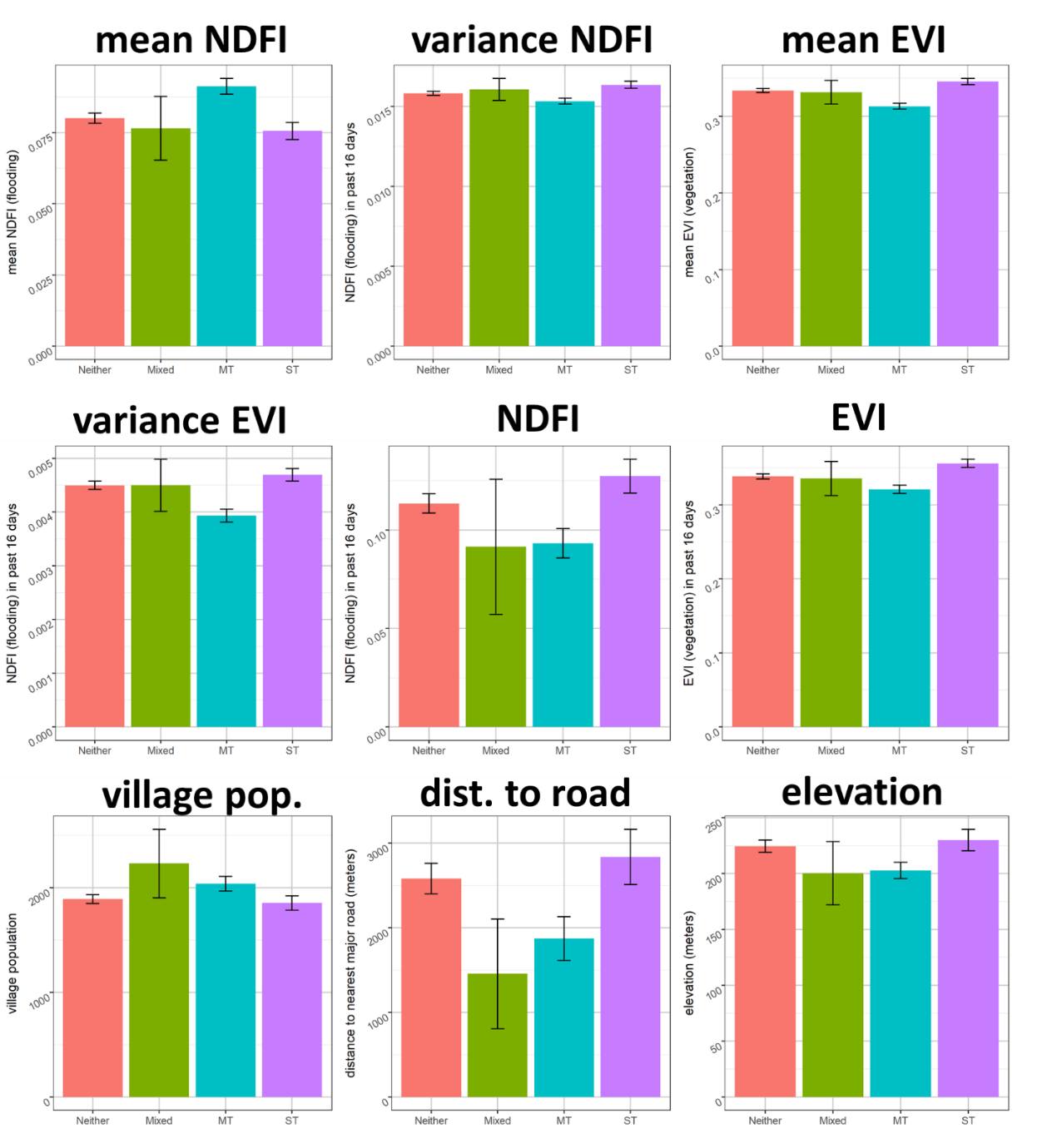

Supplement: S3 Fig — The data are split into those who were diagnosed as having scrub typhus (“ST”); murine typhus (“MT”); both ST and MT (“Mixed”); or “Neither” (indicating patients who were suspected of having typhus but were not diagnosed with either ST or MT). (TIF) [file pntd.0009685.s009.tif]

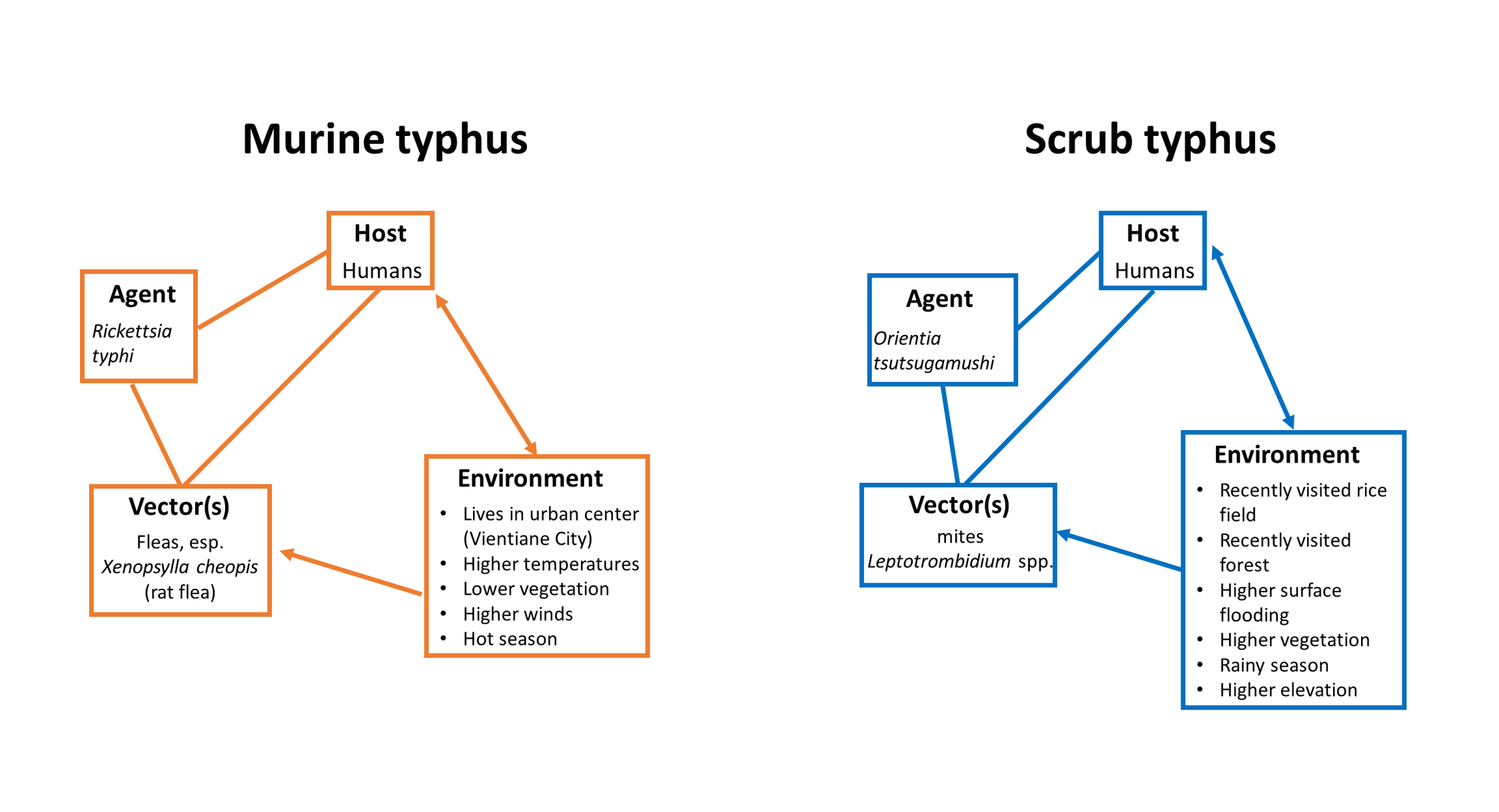

Supplement: S4 Fig — Environmental components are derived from this analysis, and some have been previously reported. We hypothesize that these environmental factors act on both vector and human populations, that humans also change the environment, and that these combinations of interactions lead to increased contact between vectors and susceptible hosts in different geographic locations and at different times (e.g. hot season for murine typhus and rainy season for scrub typhus). (TIF) [file pntd.0009685.s010.tif]
